# Evolution of clonal hematopoiesis

**DOI:** 10.1002/ctm2.1444

**Published:** 2023-10-17

**Authors:** Isabelle A. van Zeventer, Aniek O. de Graaf, Joop H. Jansen, Gerwin Huls

**Affiliations:** ^1^ Department of Hematology University Medical Center Groningen, University of Groningen Groningen Netherlands; ^2^ Department of Laboratory Medicine Laboratory of Hematology Radboud University Medical Center Nijmegen Netherlands

## 1 CLONAL HEMATOPOIESIS AS A PRE‐STAGE FOR MYELOID MALIGNANCIES

Somatic mutations and chromosomal abnormalities have a central role in the characterization, diagnosis and prognostication of myeloid malignancies. During recent years, it was discovered that hematopoietic stem and progenitor cells may also accumulate genetic aberrations with advancing age, resulting in clonal expansion of mutated cells: a process now referred to as clonal hematopoiesis (CH).^1^, ^2^ Interest in CH has markedly increased since its widespread occurrence in otherwise healthy individuals—without blood cancer—was first demonstrated in 2014.^3^, ^4^ The prevalence of CH depends on the sensitivity of the sequencing technique and increases with age. The spectrum of gene mutations is dominated by DNMT3A, TET2 and ASXL1. Different from myeloid malignancies, mutations in CH generally occur in a small percentage of blood cells as reflected by the variant allele frequency (VAF), which is the number of variant reads relative to the total number of reads at a given mutation position.^1^, ^2^ Individuals with CH have a higher risk of developing haematological malignancies, including acute myeloid leukaemia.^3^, ^5^, ^6^ Based on these findings, CH is increasingly recognized as a precursor condition for myeloid malignancies, representing an early stage in the stepwise process of clonal selection, with subsequent mutations that may result in full‐blown leukaemia.^7^

The acronym “Clonal Hematopoiesis of Indeterminate Potential” (CHIP) was first proposed to describe the presence of CH in otherwise healthy individuals, at a VAF ≥2%.^8^ CH occurs at higher frequencies when mutation screening by Next Generation Sequencing (NGS) is ordered for patients presenting with cytopenias that remain unexplained after careful evaluation and nondiagnostic bone marrow.^9^ “Clonal Cytopenia of Undetermined Significance” (CCUS) is coined to describe the presence of CH clones in such individuals with unexplained cytopenia, that do not meet established criteria for myeloid malignancy. Other proposed entities include “Clonal Monocytosis of Unknown Significance” (CMUS) for individuals carrying CH in combination with unexplained and persistent monocytosis that does not meet the diagnostic criteria for CMML. We have previously reported on the biased mutational spectra for community‐based individuals with peripheral blood count abnormalities (cytopenia or cytosis) that may raise clinical suspicion for myeloid malignancies.^10^, ^11^, ^12^ Starting in 2022, CHIP and CCUS have been formally established in WHO and ICC classifications for myeloid neoplasms, representing lower‐risk clonal lesions that may precede the development of overt malignancy.^13^, ^14^

## 2 CLONAL HEMATOPOIESIS POSES CHALLENGES FOR INTERPRETING RESULTS OF NGS MUTATIONAL SCREENING

When we detect clonal mutations in a patient with a blood count abnormality and a non‐diagnostic bone marrow, the question may be raised whether this indicates an early sign of a developing malignancy.^15^ In spite of the presence of CH, the ageing hematopoietic system mostly retains its capacity to produce the normal variety of mature blood cells. CH and cytopenia at higher ages are not necessarily related to each other: cytopenic and non‐cytopenic older individuals show considerable overlap in the mutational spectrum of CH.^10^, ^11^ However, the presence of peripheral cytopenia in addition to CH increases the risk of myeloid neoplasm development both in the general population and patients presenting in the clinic. The diagnostic work‐up should thus distinguish individuals with high‐risk CH from those with innocuous age‐related clones that have little or no risk of malignancy.

Clonal dynamics, external pressures and progression to malignancy.

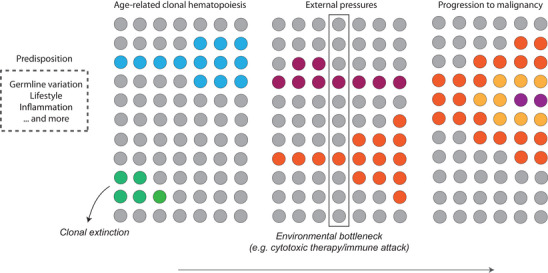

Graphical illustration of clonal expansion potential and risk of malignancy development associated with recurrent gene mutations in the general population. Purple, DNA damage response genes; green, RNA splicing; blue, epigenetic regulators (including DNA methylation and histone modification); red, signal transduction.

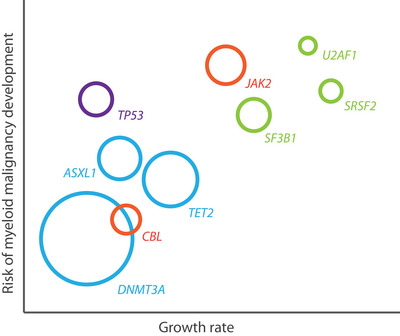

## 3 EVOLUTION OF CLONAL HEMATOPOIESIS IN THE GENERAL POPULATION

Knowledge about the clonal dynamics over time and selective pressures that may drive clonal expansion is crucial for our understanding of the natural history of CH. The first studies on mutational data generated from longitudinal sampling have recently been published. Gene‐specific fitness effects determining clonal outgrowth were shown in the Lothian Birth Cohort.^16^ Fabre et al. further tracked gene‐specific clonal dynamics in peripheral blood samples from 385 adults in the Sardinia longitudinal study.^17^ We recently reported growth rates for common myeloid driver gene mutations based on sequential VAF measurements in 3359 individuals ≥60 years from the population‐based Lifelines cohort.^18^ These studies show that DNMT3A and TP53‐mutated clones are characterized by very limited clonal expansion, whereas highest growth rates are observed for clones with mutations in the splicing factor genes (SF3B1, SRSF2 and U2AF1) and JAK2. TET2 and ASXL1 mutated clones show intermediate expansion potential. The dynamics of clonal expansion are independent of peripheral blood cytopenia or cytosis, which were present in 39% of individuals in our study cohort.^18^ Gene mutations in CH have a frequency distribution different from myeloid cancers: NPM1, EZH2, KRAS/NRAS, RUNX1 or IDH1/2 mutations are rarely detected in the general population. Using the large‐scale longitudinal data, we show that such mutations are typically acquired late in the evolution of CH, co‐occurring with so‐called “founder” mutations.^18^

## 4 HIGH‐RISK MUTATIONAL SPECTRA

Next to the presence of peripheral blood cytopenia or cytosis, increased clonal expansion, reflected by a higher clone size or VAF, has been associated with myeloid neoplasm risk and adverse health events across numerous studies.^3^, ^4^, ^5^, ^6^, ^9^, ^18^, ^19^, ^20^, ^21^ Several other mutational markers of CH have prognostic relevance. Spliceosome gene mutations, JAK2, as well as more rarely detected genes (including RUNX1, EZH2, KRAS, NRAS, IDH1 and IDH2) invariably predict a higher risk of myeloid malignancies among individuals with CHIP and CCUS. In addition, co‐mutational spectra involving multiple gene mutations are associated with increased progression risks.^5^, ^6^, ^9^, ^18^, ^19^, ^20^, ^21^, ^22^, ^23^ These mutational spectra also characterized the deviating mutational spectra for population‐based subjects in the context of blood count abnormalities.^10^, ^11^, ^12^, ^18^ JAK2 mutations should raise suspicion for myeloproliferative neoplasm pre‐stages and may facilitate early recognition, especially in the context of peripheral blood cytosis.^11^, ^18^ Interestingly, DNMT3A mutations are most frequently detected in the general population but have no impact on blood counts or the development of myeloid malignancies. Other markers of prognostic relevance include mean corpuscular volume (MCV) and red blood cell distribution width (RDW).^6^, ^20^ Finally, the joint role of gene mutations and clonal chromosomal abnormalities in haematological malignancy development warrants further study.^24^

## 5 ‘GENOTYPE (G) + ENVIRONMENT (E) = PHENOTYPE (P)’

Apart from gene‐specific trajectories, there is still considerable inter‐individual variation in growth trajectories, even when such individuals carry the exact same mutation. Bona fide driver mutations do not always lead to considerable expansion,^17^, ^18^ implicating that extrinsic factors that stimulate or dampen clonal expansion play an important role. Several stressors that may drive clonal expansion have been identified. For example, TP53 mutated clones show preferential expansion under cytotoxic stress^25^, ^26^ and specific mutations (including BCOR/BCORL1) may escape the auto‐immune attack in patients with aplastic anaemia.^27^ Traditional cancer risk factors, including smoking, sex and excessive alcohol use, did not affect overall clonal expansion in the Lifelines cohort.^18^ However, ample evidence for a relation between ASXL1 mutations and smoking^26^, ^28^, ^29^ suggests that the role of such factors on the dynamics of specific gene mutations needs further exploration. Extended longitudinal data are needed to further define intrinsic and extrinsic factors supporting or hampering clonal outgrowth and progression to malignancy, for example including germline constitution and (age‐related) inflammatory insults.

## 6 CONCLUSION

Recent work has refined our knowledge about the dynamics of clonal hematopoiesis in healthy individuals. Driver mutations are important determinants in the trajectory of clonal evolution—both in terms of expansion and risk of incident malignancy—and should be used to define a consensus spectrum of high‐risk CHIP or CCUS. Identification of extrinsic factors that can modulate clonal expansion may provide rationales for intervention studies.

## CONFLICT OF INTEREST STATEMENT

The authors declare no conflict of interest.
